# Engineering Properties and Microscopic Mechanisms of Permeable and Flexible Polymer-Improved Sand

**DOI:** 10.3390/polym17131856

**Published:** 2025-07-02

**Authors:** Yang Zeng, Yongli Xie, Jiaxiang Liu

**Affiliations:** School of Highway, Chang’an University, Xi’an 710064, China

**Keywords:** polymer, sand layer, grouting, mechanical performance, microstructure

## Abstract

Grouting is an effective method for enhancing the stability of poor strata such as sand layers. The performance of the grouting materials directly influences the effect of stratum reinforcement. To meet the urgent demand for efficient grouting materials, this study selected a high-permeability, flexible polymer (PFP) as the grouting material. The influences of the PFP content, curing time, and dry density on the mechanical and impermeable properties of PFP-improved sand were systematically analyzed via unconfined compressive tests, split tensile tests, and variable head permeability tests. Moreover, the section morphology and pore characteristics of the PFP-improved sand were qualitatively described and quantitatively analyzed by scanning electron microscopy (SEM) and image processing software. The results indicated that the mechanical properties and impermeability of the test sand were significantly improved by adding the PFP, and the improvement effect continued to increase with increasing PFP content, curing time, and dry density. The compressive strength and splitting tensile strength of PFP30 (PFP content of 30%, curing time of 28 d, dry density of 1.5 g/cm^3^) reached 8.3 MPa and 1.4 MPa, respectively. The permeability coefficient reduced to 5.41 × 10^−6^ cm/s. The microscopic results revealed that the PFP effectively cemented the isolated sand particles through bridging, filling, and encapsulation as well as substantially filled the internal pores of the test sand. The percentage of the pore area, the total number of pores, and the maximum pore diameter of the test sand were significantly reduced. The pore area percentage, the total number of pores, and the maximum pore diameter of PFP30 were reduced to 0.124, 30, and 213.84 μm, respectively. This study reveals that PFP has potential for application in the grouting construction of poor strata, such as sand layers.

## 1. Introduction

Sand layers are undesirable strata frequently encountered in engineering construction, and they are widely distributed in coastal, riverine, and plain areas [1]. Due to their poor cementation, loose structure, low bearing capacity, and ease of being disturbed, sand layers are very prone to deformation and instability during construction [2,3,4]. Therefore, it is of great significance to improve the mechanical properties of sand layers to ensure the safety of tunnel construction. Among the many reinforcement methods, the grouting method is widely used in the reinforcement of poor strata due to its advantages of having a short cycle, having remarkable effects, and being a simple process [5,6,7].

Currently, the prevailing grouting materials mainly include cement-based materials, chemical materials and biopolymer-based grouting materials [8,9,10,11]. As traditional grouting materials with a long history of application, cement-based materials have advantages such as low cost, non-toxicity, a wide source of raw materials, a simple grouting process, and high strength in stone structures [12,13,14]. However, because cement-based materials are granular materials, cement particles, when injected, are extremely restricted by the soil structure in the process of grouting diffusion, meaning its actual diffusion range is relatively limited [15]. And studies have found that the surrounding rock after the grouting of cement-based materials is mostly rigid and brittle. Reinforced perimeter rock is highly susceptible to cracking and sudden damage induced by external factors (construction disturbances, freeze–thaw cycles, dry–wet alternation, etc.) [16,17].

Compared to cement-based materials, chemical-based materials have the advantages of a controllable gel time, pumpability, and the ability to penetrate voids. However, the commonly used chemical slurries usually have problems such as high brittleness, uncontrollable shrinkage, high viscosity, and environmental pollution [18,19,20]. Biopolymer-based grouting materials have the advantages of low energy consumption, low cost, and being environmentally safe, but they still face limitations such as the uneven distribution of calcium carbonate, and they are greatly influenced by the environment [21,22,23]. Therefore, an efficient grouting material with excellent permeability, considerable strength, and environmental friendliness must be developed.

Zeng et al. [24] researched a new type of permeable flexible polymer grouting material (PFP), which was confirmed to have excellent permeability, strength, and flexibility through systematic experiments. Further grouting comparison experiments showed that the PFP was significantly better than traditional grouting materials in terms of diffusion radius, permeability uniformity, and consolidation effect. It showed good potential for engineering applications. However, the study did not systematically analyze the evolution of key indicators such as the mechanical properties (compressive strength and tensile strength) and hydrological properties (permeability coefficient) of the improved sand under gradient changes in PFP content and different curing times. The evolution laws of key parameters such as porosity, pore size, and pore number within the PFP-improved sand were also not quantitatively characterized. This lack of correlation between the macroscopic mechanical properties and microstructural features means that the relationship between the modification mechanism of PFP and the macroscopic properties of its reinforced sand has not been fully explained.

In order to systematically study the improvement effect of PFP on the engineering properties of sand layers, this study examined the effects of PFP content and curing time on the engineering properties of test sand at different dry densities by using an unconfined compression test, split tensile test, and variable head penetration test. The microstructure and pore characteristics of the improved sand were qualitatively described and quantitatively analyzed by SEM and image recognition software (Matlab2025). This study provides a theoretical foundation and research basis for the field application of PFP grouting materials.

## 2. Materials and Methods

### 2.1. Sand

**Figure 1:**
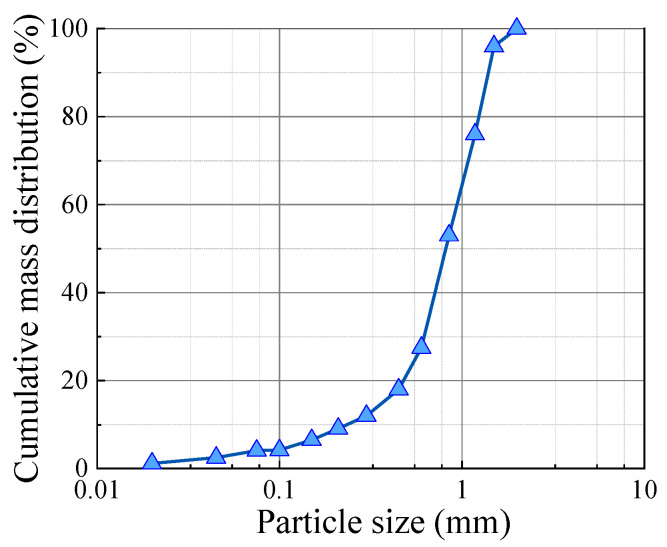
Grain gradation curves of test sand.

We followed the Standard for Geotechnical Testing Method (GB/T 50123-2019) [25]. Test sand was dried, and particles less than 2 mm in size were retained to simulate the sand layer for the tests. A cumulative curve of the grain size grading of the test sand is shown in Figure 1. The uniformity coefficient Cu of the test sand was 3.79, and the curvature coefficient Cc was 1.68, which indicated poorly graded sand. The test parameters of the test sand are shown in Table 1.

### 2.2. Grouting Materials

**Figure 2:**
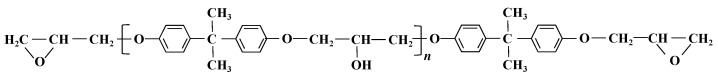
Molecular structure of component A.

**Figure 3:**
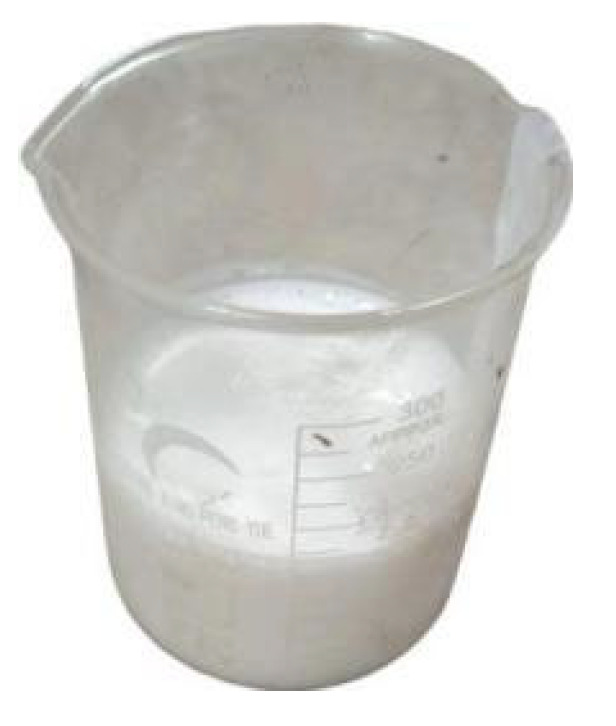
PFP grouting material.

The grouting material (PFP) can provide adhesion for soil particles after solidification, which can effectively improve the strength of the soil. The PFP components included components A, B, C, and D. Component A was an epoxy resin (Model ZKAT-101). Component B was a modified amine curing agent (Model ZKAT-L301). Component C was the diluent, and the composition was water. Component D was a coagulant that was composed of dimethyl phthalate. The chemical structure of the epoxy resin is shown in Figure 2. The epoxy groups on both sides of the epoxy resin resulted in good reactivity and could react with the curing agent. The PFP grouting material is shown in Figure 3, and its basic physical parameters are given in Table 2.

### 2.3. Experimental Procedure

#### 2.3.1. Specimen Preparation

The ratio of PFP to dry sand quality was defined as the PFP content. Different contents of PFP were mixed with the test sand, poured into standard molds, and pressed to the target dry density (1.40 g/cm^3^, 1.45 g/cm^3^, and 1.50 g/cm^3^) by applying a static load. The specimen used for the mechanical test was a cylindrical sample with a height of 80 mm and a diameter of 39.1 mm. The specimens used for the variable head permeability test had a height of 40 mm and a diameter of 61.8 mm and were round and cake-shaped.

**Figure 4:**
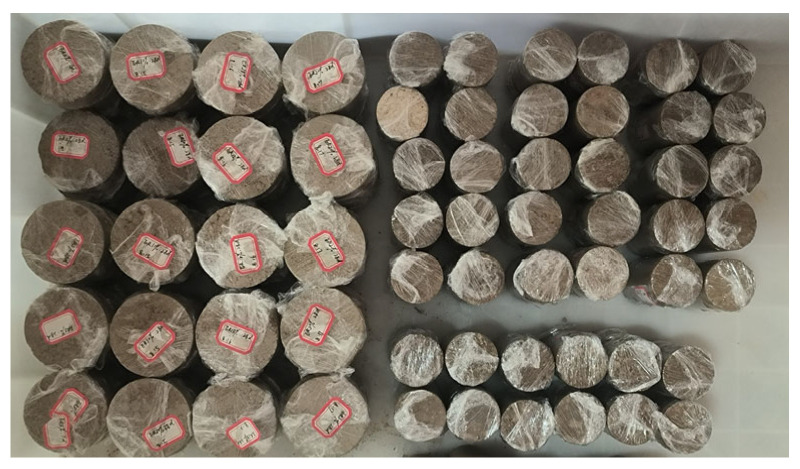
Partial test specimen.

Before formal specimen preparation, the PFP content was set at 5%, 10%, 15%, 20%, 25%, 30%, 35%, and 40% for pretesting. The samples were very easy to break and difficult to remove completely when the PFP content was 5% or 10%. The reason for this could be that when the PFP content was low and the setting time was short, the bond strength between sand particles was low, so the samples were very easily broken under the influence of external forces during demolding. The samples were significantly fluid–plastic when the PFP content was 35% or 40%, and the samples were difficult to compact to the target density. Therefore, the PFP mixing range was set to 15%, 20%, 25%, and 30%, and the samples were grouped as shown in Table 3. Each sample was left to stand for one day and then demolded, and the samples were cured in a constant environment at 25 °C and 80% humidity until reaching the specified age. Some of the test samples are shown in Figure 4.

#### 2.3.2. Mechanical Property Testing

The samples were removed after curing them to the specified age; unconfined compressive strength tests were carried out. The press model was YAW-3000, and the loading rate was 1.5 kN/s. The model of the press used for the split tensile test was YAW-3000. A cylindrical sample was placed horizontally between two horizontal loading plates. A rigid pad was placed between the sample and the loading plate at the contact point, which transmitted a linearly distributed pressure load to the cylindrical sample. Under pressure, tensile stress was generated along the vertical surface of the sample until it fractured. The pressing rate was 1.5 kN/s until the sample was damaged. Considering the possible plastic deformation of the samples before fracture damage, the splitting tensile strength value of each sample was calculated via the modified splitting tensile strength formula [26]. The compressive strength results were taken as the average of three sets of parallel tests.

(1) $$\sigma =\frac{2P}{\pi dL}g(x)$$

In the formula, *σ* is the tensile strength; *P* is the magnitude of radial load; *d* is the diameter of the cylindrical specimen; *L* is the total length of the specimen; and the expression for the correction factor *g(x)* is

(2) $$g(x)=-\frac{d}{2a}\left\{2f-sin\left(2f\right)-\frac{{2y}_{1}}{d}lg\left(tan\left(\frac{\pi }{4}+\frac{f}{2}\right)\right)\right\}$$

In the formula, *a* is the half-width of the flattened area; *d* is the diameter of the cylindrical specimen; *y*_1_ is the distance between the flattened surfaces when 1/2 of the specimen is destroyed; *f* is the flattening rate, *f = a/y*_1_.

#### 2.3.3. Variable Head Permeability Test

The permeability of the specimens was tested by the variable head permeability test method with a model TST-55 instrument. The permeability coefficients were calculated according to Formulas (3) and (4):

(3) $$k_{T}=2.3\frac{aL}{At}lg\frac{H_{b1}}{H_{b2}}$$

(4) $$k_{20}=k_{T}\frac{\eta_{r}}{\eta_{20}}$$

In the formulas, *a* is cross-sectional area of the variable head pipe (cm^2^); *A* is the cross-sectional area of the specimen (cm^2^); *L* is the penetration path (cm); *H_b_*_1_ is the starting water head (cm); *H_b_*_2_ is the ending water head (cm).

#### 2.3.4. SEM Test

After the mechanical experiments, typical block samples were selected for SEM to analyze the effect of the PFP on the microstructure of the test sand. The scanning electron microscope used was a Sigma 300 model manufactured by Zeiss in Oberkochen, Germany.

#### 2.3.5. Image Processing and Quantitative Analysis

**Figure 5:**
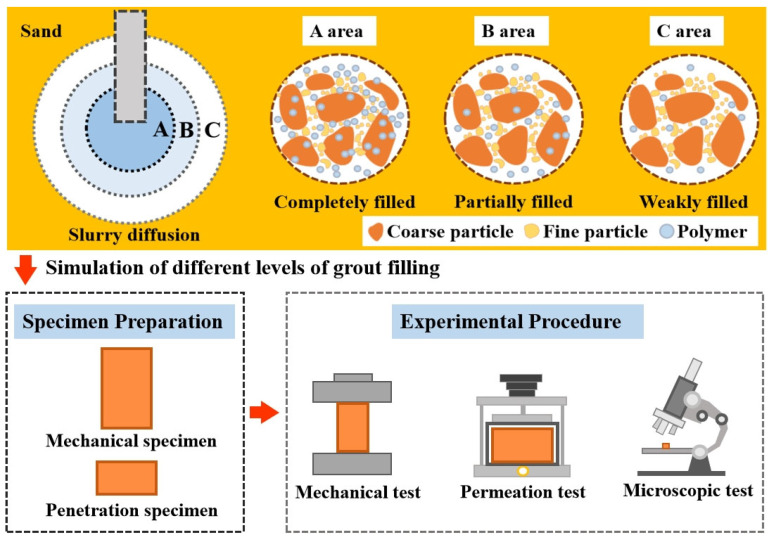
Total experimental procedure.

To quantitatively compare the influence of different PFP contents on the microstructure of the test sand, SEM images of grouted sand were analyzed via image processing software. First, the original images were converted into grayscale images and then binarized by grayscale thresholding. The image processing software automatically determined the sample area, pore area percentage, and other pore diameters. The total experimental procedure is shown in Figure 5.

## 3. Results

### 3.1. Unconfined Compressive Strength and Splitting Tensile Strength of PFP-Improved Sand

**Figure 6:**
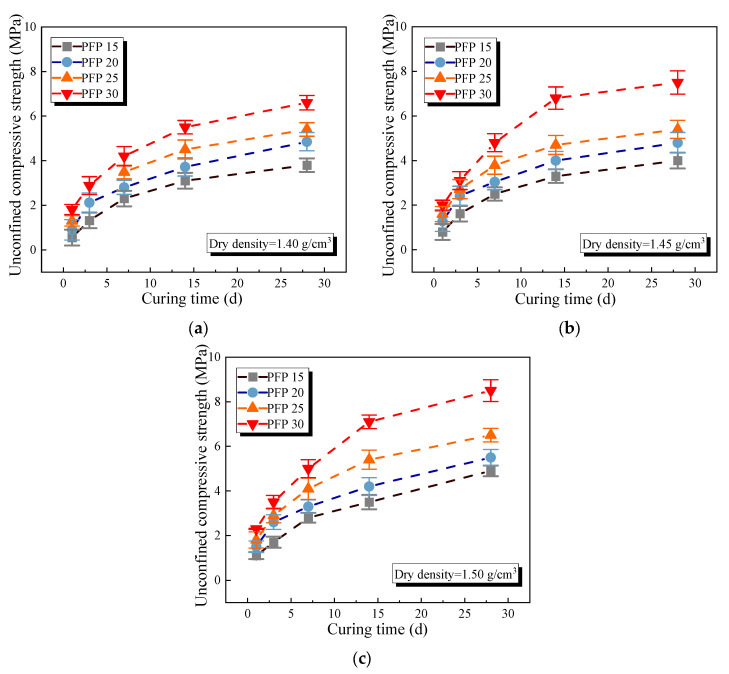
Unconfined compressive strength of PFP-improved sand: (**a**) dry density 1.40 g/cm^3^; (**b**) dry density 1.45 g/cm^3^; (**c**) dry density 1.50 g/cm^3^.

**Figure 7:**
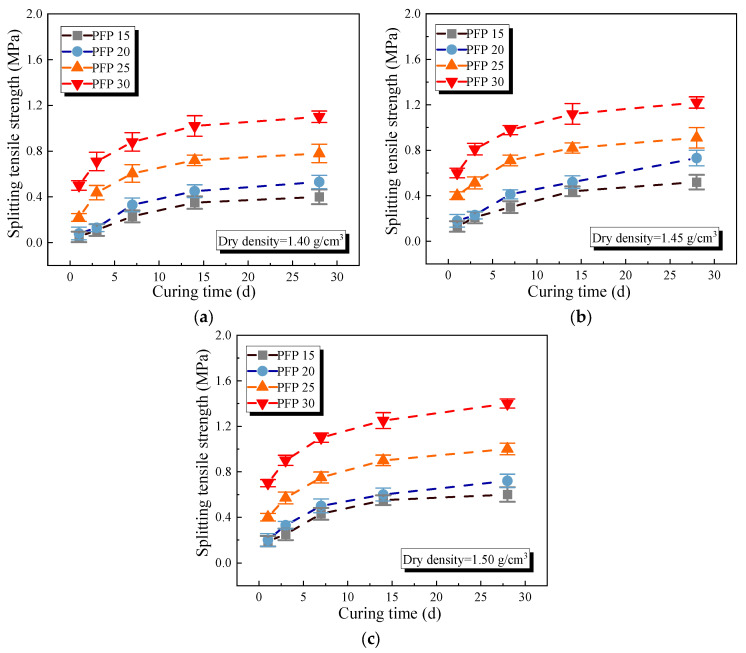
Splitting tensile strength of PFP-improved sand: (**a**) dry density 1.40 g/cm^3^; (**b**) dry density 1.45 g/cm^3^; (**c**) dry density 1.50 g/cm^3^.

Figure 6 and Figure 7 show the unconfined compressive and tensile strength curves of the improved sand with different PFP contents. The mechanical properties of the PFP-improved sand tended to increase logarithmically with increasing PFP content. At a curing age of 1 d and a dry density of 1.40 g/cm^3^, the compressive strengths of PFP15, PFP20, PFP25, and PFP30 reached 0.59 MPa, 0.88 MPa, 1.31 MPa, and 1.82 MPa, respectively, and the tensile strengths were 0.06 MPa, 0.08 MPa, 0.22 MPa, and 0.51 MPa, respectively. An increase in the curing time, and dry density positively contributed to the strength of the PFP-improved sand. When the curing time increased to 28 d, the dry density increased to 1.50 g/cm^3^. The peak compressive strengths of PFP15, PFP20, PFP25, and PFP30 reached 4.25 MPa, 4.31 MPa, 4.98 MPa, and 8.18 MPa, respectively. The peak tensile strengths reached 0.61 MPa, 0.72 MPa, 1.03 MPa, and 1.44 MPa, respectively.

The improvement mechanism of PFP on the sand’s mechanical properties could be attributed to two aspects. First, due to the filling effect, the pores of the test sand were filled with PFP, greatly improving the degree of compactness of the test sand. Second, because of the bonding effect, PFP provided an effective binding force to the sand particles within the solidification range, limiting the displacement of the sand particles. The isolated sand particles gradually formed agglomerates under the three-dimensional network connection formed by PFP solidification. With increasing curing time, the epoxy resin molecules and curing agent molecules in the PFP slurry gradually reacted. As a result, the filling and bonding effects of PFP became increasingly significant, and its strength increased. The dry density affected the strength of a sample lies because, at lower dry densities, the sand sample was loosely structured with large pores between the sand particles. This was favorable for the encapsulation of sand particles by PFP, but, accordingly, the linkage strength of the PFP between sand particles was relatively weak. When the density of the sand sample was high, the contact between the sand particles became close. On the one hand, it increased the friction strength between sand particles, and, on the other hand, it strengthened the degree of pore filling of the sample.

### 3.2. Permeability of PFP-Improved Sand

**Figure 8:**
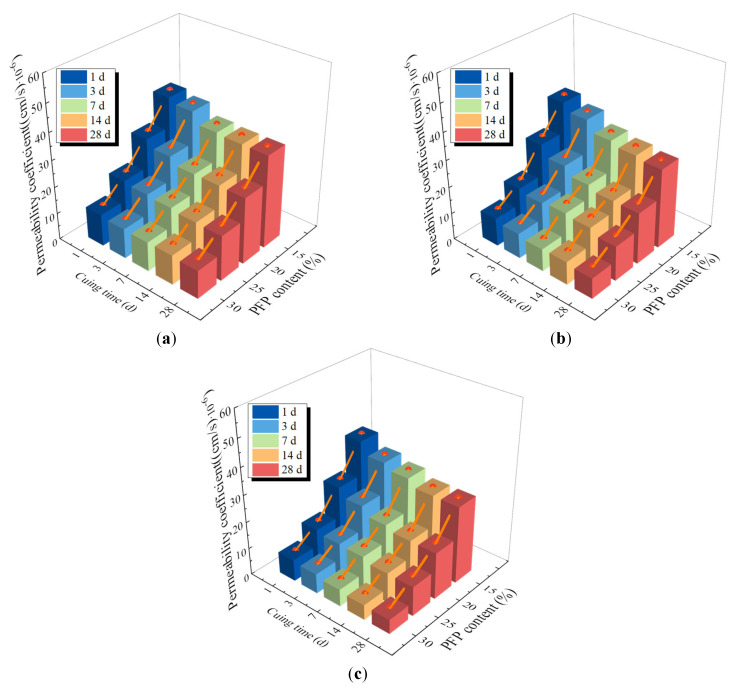
Permeability coefficient of PFP-improved sand: (**a**) dry density 1.40 g/cm^3^; (**b**) dry density 1.45 g/cm^3^; (**c**) dry density 1.50 g/cm^3^.

Figure 8 shows the permeability coefficient of the improved sand with different PFP contents. As shown in Figure 8, the permeability coefficient of the improved sand decreased with increasing PFP content. The permeability coefficient of the PFP-improved sand decreased from 37.51 × 10^−6^ cm/s (PFP15) to 6.43 × 10^−6^ cm/s (PFP30) when the curing time was 1 d and the dry density was 1.50 g/cm^3^, a decrease of approximately 90%. The permeability of the improved sand effectively improved. This was attributed to the filling effect of the PFP on the internal pores of the test sand. The PFP had low viscosity and good fluidity, so the large, medium, and small pores inside the test sand were effectively filled. The degree of compactness of the test was closely related to the content of PFP. When the PFP content was high, the degree of compactness of the PFP-improved sand was greater.

The permeability coefficients of the PFP-improved sand decreased and then stabilized with increasing curing age. The permeability coefficients of PFP15, PFP20, PFP25 and PFP30 decreased from 37.51 × 10^−6^ cm/s cm/s, 25.13 × 10^−6^ cm/s cm/s, 14.07 × 10^−6^ cm/s, and 8.06 × 10^−6^ cm/s (curing time of 1 d) to 28.62 × 10^−6^ cm/s, 17.35 × 10^−6^ cm/s, 11.05 × 10^−6^ cm/s, and 5.41 × 10^−6^ cm/s (curing time of 28 d), respectively. The permeability coefficients of the PFP-improved sand with different PFP contents basically stabilized at a curing time of 7 d. This may have been due to the low bond strength of the PFP in the early stage of the curing reaction, resulting in weak interfacial connections between sands. The pore structure and connectivity of the PFP-improved sand altered under seepage. With increasing curing time, the PFP reached a certain strength, and the adhesion effect between the sand particles was stable, so the permeability coefficient of the PFP-improved sand gradually tended to stabilize. The permeability coefficient of the PFP-improved sand slightly decreased with increasing dry density. This is because the permeability of soil is strongly affected by the number of large- and medium-sized pores and the thickness of the pore channels. An increase in the dry density of the sample made the sand particles more compact, the pore size reduced, and the pore channels were narrower and tortuous. Therefore, the permeability coefficient of the sample with a dry density of 1.50 g/cm^3^ was the lowest (5.41 × 10^−6^ cm/s).

### 3.3. Correlation Analysis

**Figure 9:**
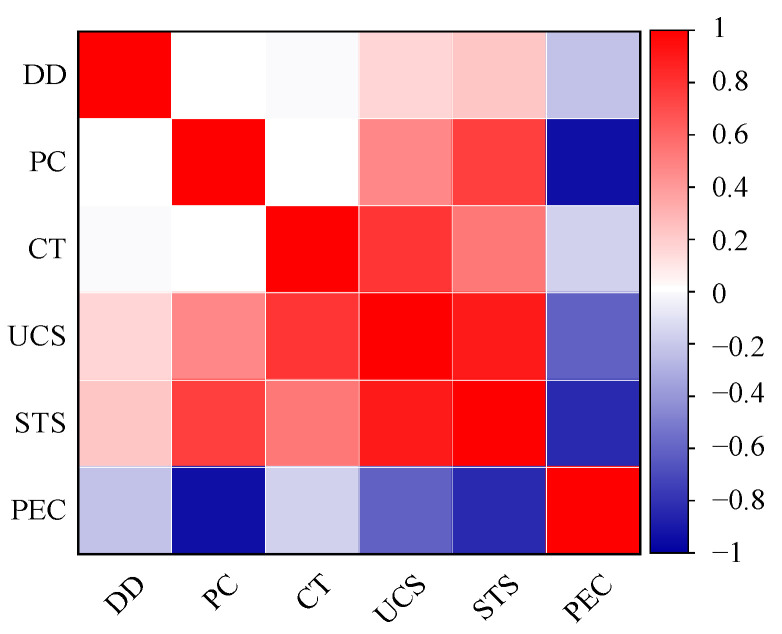
Thermogram of correlation of different factors with the strength and permeability coefficient of PFP-improved sand.

On the basis of the above experimental results, correlation analyses of the PFP content (PC), curing time (CT), and dry density (DD) with the compressive strength (UCS), split tensile strength (STS) and permeability coefficient (PEC) of the improved sand were carried out, and the results are shown in Figure 9. The mechanical properties of the improved sand (compressive strength and splitting tensile strength) positively correlated with the PFP content, curing time, and dry density, with a significant correlation with the curing time and PFP content and a weak correlation with the dry density. The permeability coefficient of the improved sand negatively correlated with the PFP content, curing time, and dry density, which indicated that the impermeability of the improved sand increased with increasing PFP content, curing time, and dry density.

### 3.4. Micromechanisms of PFP-Improved Sand

#### 3.4.1. SEM of PFP-Improved Sand

**Figure 10:**
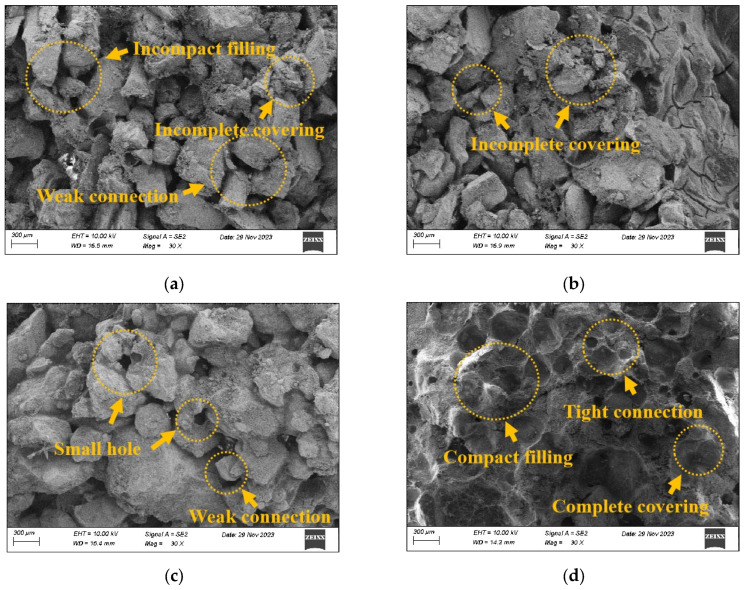
Microstructure of PFP-improved sand: (**a**) PFP15; (**b**) PFP20; (**c**) PFP25; (**d**) PFP30.

To analyze the micromechanisms of the engineering properties of PFP-improved sand in detail, PFP-improved sands with a curing age of 28 d and a dry density of 1.50 g/cm^3^ were selected for microscopy experiments. Figure 10 shows the microscopic morphology of the improved sand with different PFP contents. Figure 10 shows that the internal pores of PFP15 filled to a relatively small extent, and the sand particles were mostly in point contact with each other. The number of pores and cracks in PFP15 was relatively high and variable in size. The PFP coating layer on the surface of the sand particles was weak, and the coating area was not large. PFP did not form effective bonding between the sand particles. With increasing PFP content, the microstructure of the improved sand changed significantly. The microscopic morphology of PFP30 revealed that the degree of structural integrity was high. The microfractures and cavities were filled by PFP, and the contact mode between the sand particles changed from point contact to surface contact. The contact area and degree of occlusion between the sand particles significantly increased. After condensation, PFP showed a three-dimensional network structure, which adhered tightly on the surface of the sand particles, and the surface of the sand particles was covered by PFP. The loose sand particles thus formed a large PFP–sand particle agglomerate. Under the constraints of the PFP network, the displacement of the sand particles caused by the external load was effectively limited. The addition of PFP changed the internal structure of the test sand from loose to dense, its degree of homogeneity was greatly improved, and there were no obvious pores or cracks. The loose structure of the test sand was effectively improved by the bonding and filling effects of the PFP. The integrity of the improved sand significantly increased, and the bearing capacity effectively improved. With increasing PFP content, the improvement was more significant.

#### 3.4.2. Pore Parameters of PFP-Improved Sand

**Figure 11:**
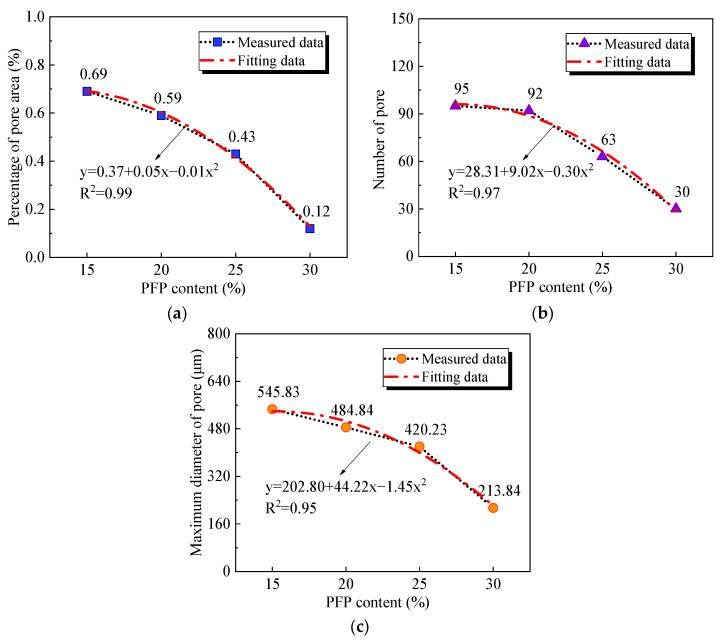
Pore parameters of PFP-improved sand: (**a**) pore area percentage; (**b**) number of pores; (**c**) maximum diameter of pores.

**Figure 12:**
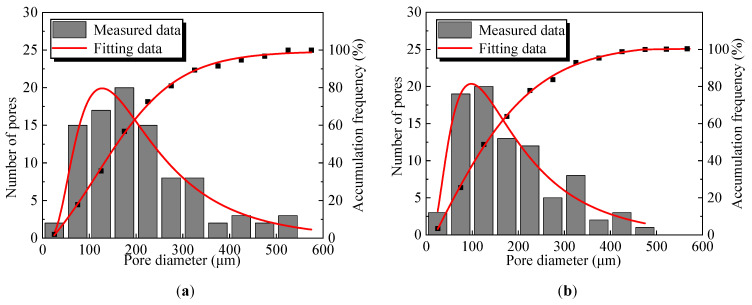

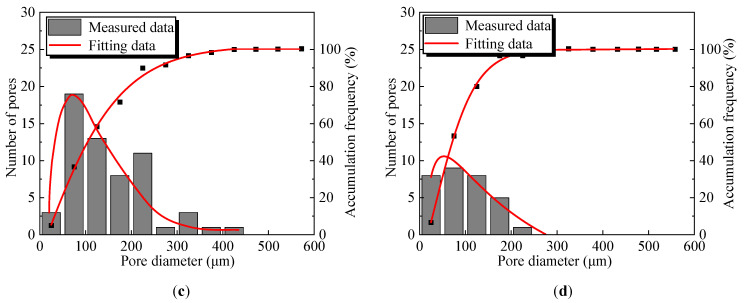
Pore length distribution of PFP-improved sand: (**a**) PFP15; (**b**) PFP20; (**c**) PFP25; (**d**) PFP30.

Figure 11 shows the pore area percentage, the number of pores, and the maximum pore diameter of the improved sands with different PFP contents. The three pore parameters decreased quadratically with increasing PFP content. The pore area percentage, overall pore number, and maximum pore diameter of PFP15 were 0.69, 95, and 545.83 μm, respectively. In contrast, the pore area percentage, the number of pores, and maximum diameter of the pores in PFP30 were reduced to 0.12, 30, and 213.84 μm, respectively. Figure 12 shows the pore diameter distributions of the four groups of samples. According to the pore diameter distribution law, with increasing PFP content, the pore distribution range of the PFP-improved sand gradually narrowed. The differences in the pore parameters of the four groups of samples indicated that the addition of PFP significantly improved the internal defects of the test sand. The pores of the test sand gradually filled with PFP. The degree of pore bond filling increased with increasing PFP content. The pores of the sample tended to be homogeneous, so the pore area percentage of the sample decreased significantly, the overall number of pores decreased significantly, and the maximum pore diameter was effectively controlled. Since the pores had a significant effect on the strength and permeability properties of the sand, this fully validated the improvement in the mechanical and permeability properties of the improved sand with increasing PFP content.

## 4. Conclusions

In this work, a self-developed highly permeable, flexible polymer (PFP) was used as a grouting material to improve sand at different dry densities. The relationships among the strength and permeability coefficient of the improved sand with different PFP contents, curing times, and dry densities were investigated via mechanical and permeability experiments. Finally, SEM and image processing techniques were combined to analyze the evolution of the internal pore parameters of the sand after PFP injection, which revealed the microimprovement mechanisms of the PFP in the test sand.

The PFP content, curing time, and dry density positively correlated with the improvement in the mechanical properties of the tested sands. The compressive strength and split tensile strength of the samples were the highest when the PFP content was 30%, the age of maintenance was 28 d, and the dry density was 1.50 g/cm^3^. The compressive strength and split tensile strength of PFP30 were 8.3 MPa and 1.4 MPa, respectively.

The permeability of the PFP-improved significantly with increasing PFP content, curing time, and dry density. The increase in PFP content produced the most significant improvement in the permeability of the improved sands, followed by the dry density, and the lowest curing time. When the PFP content was 30%, the dry density was 1.50 g/cm^3^, the curing time was 28 d, the sample was the most impermeable, and the permeability coefficient reduced to 5.41 × 10^−6^ cm/s.

The PFP effectively strengthened the contact between isolated sand particles through bridging, filling, and encapsulation mechanisms. The isolated sand particles effectively bonded into agglomerates by the PFP. The integrity of the test sand was greatly improved. The pore area percentage, overall pore number, and maximum pore diameter of the test sand were effectively reduced. With increasing PFP content, the improvement effect was more significant. When the PFP content reached 30%, the pore area percentage, overall pore number, and maximum pore diameter of the test sand reduced to 0.124, 30, and 213.84 μm, respectively.

## Figures and Tables

**Table 1:** Parameter specifications of test sand.

Test Parameters	Density (kg·m^−3^)	Porosity (%)	Coefficient of Uniformity Cu	Coefficient of Curvature Cc
Test sand	1410	41	3.79	1.68

**Table 2:** Physical properties of PFP grouting material.

Characteristics	Density (g/cm^3^)	Viscosity (20 °C, mPa·s)	Setting Time (min)	Compressive Strength (28 d, MPa)
Milky white liquid	0.98	19.61	200–220	12

**Table 3:** Indoor test design for PFP-improved sand.

Specimen Number	PFP Content (%)	Curing Time (d)	Dry Density (g/cm^3^)
PFP15	15	1, 3, 7, 14, 28	1.40, 1.45, 1.50
PFP20	20	1, 3, 7, 14, 28	1.40, 1.45, 1.50
PFP25	25	1, 3, 7, 14, 28	1.40, 1.45, 1.50
PFP30	30	1, 3, 7, 14, 28	1.40, 1.45, 1.50

## Data Availability

The data present in this study are available upon request.
